# Artificial Intelligence–Driven Hypertension Management: Implications for Quality Improvement and Prevention of End-Organ Damage

**DOI:** 10.3390/life16040573

**Published:** 2026-04-01

**Authors:** Laura Ramlawi, Serge Sicouri, Vasiliki Androutsopoulou, Massimo Baudo, Andrew Xanthopoulos, Alexandra Bekiaridou, Dimitrios E. Magouliotis

**Affiliations:** 1 Department of Science, Marianopolis College, Westmount, QC H3Y 1X9, Canada; laura.ramlawi@gmail.com; 2 Department of Cardiac Surgery Research, Lankenau Institute for Medical Research, Main Line Health, Wynnewood, PA 19096, USA; massimo.baudo@icloud.com (M.B.); magouliotisd@mlhs.org (D.E.M.); 3 Department of Cardiothoracic Surgery, University of Thessaly, Biopolis, 41110 Larissa, Greece; androutsopoulouvasiliki@uth.gr; 4 Department of Cardiology, University of Thessaly, Biopolis, 41110 Larissa, Greece; andrewvxanth@gmail.com; 5 Elmezzi Graduate School of Molecular Medicine, Northwell Health, Manhasset, NY 11030, USA; ampekiaridou@gmail.com

**Keywords:** artificial intelligence, hypertension management, quality improvement, end-organ damage, primary care

## Abstract

Hypertension remains a leading modifiable risk factor for cardiovascular morbidity and mortality. Nonetheless, blood pressure control rates remain suboptimal despite established treatment guidelines and effective pharmacologic therapies. In parallel, artificial intelligence (AI) has rapidly expanded within cardiovascular medicine, demonstrating promising capabilities in disease detection, risk prediction, and clinical decision support. However, most AI applications in hypertension have focused primarily on algorithmic performance rather than real-world implementation or measurable improvements in patient outcomes. This review examines artificial intelligence-driven hypertension management through the lens of quality improvement and prevention of end-organ damage. We summarize current applications of machine learning, deep learning, natural language processing, and imaging analytics in hypertension detection and risk stratification, and critically evaluate their integration into clinical workflows. Particular emphasis is placed on therapeutic inertia, primary care-centered implementation, and the use of AI to support continuous quality improvement frameworks. Beyond blood pressure reduction alone, we explore the potential of AI to identify patients at risk for hypertensive heart disease, heart failure, aortic pathology, renal dysfunction, and cerebrovascular events. We discuss implementation challenges, including external validation, algorithmic bias, workflow integration, and regulatory considerations, which must be addressed to ensure safe and equitable deployment. Artificial intelligence offers the opportunity to transform hypertension management from reactive blood pressure control to proactive organ protection. Critically, AI-driven quality improvement interventions must be evaluated against established non-AI strategies, including pharmacist-led management and team-based care, which provide the benchmarks for demonstrating added clinical value. Achieving this shift will require embedding predictive analytics within structured, outcome-oriented systems of care and rigorously evaluating their impact on cardiovascular morbidity and mortality.

## 1. Introduction

Hypertension remains one of the most prevalent and modifiable risk factors for cardiovascular morbidity and mortality worldwide [1]. Despite the availability of effective pharmacologic therapies and well-established clinical guidelines, blood pressure (BP) control rates remain suboptimal in the United States and globally [2]. Persistent gaps in detection, treatment intensification, and longitudinal monitoring continue to limit the real-world effectiveness of hypertension management strategies.

Contemporary hypertension guidelines emphasize early diagnosis, risk-based treatment thresholds, and structured follow-up to prevent adverse cardiovascular outcomes, including myocardial infarction, stroke, heart failure, and vascular disease [3]. However, translating these recommendations into consistent clinical practice remains challenging, particularly in primary care settings where the majority of hypertension diagnosis and long-term management occurs [4]. Therapeutic inertia, fragmented care delivery, workflow constraints, and inconsistent adherence to evidence-based protocols contribute substantially to ongoing uncontrolled hypertension [5].

Over the past decade, artificial intelligence (AI) and machine learning (ML) techniques have been increasingly applied to cardiovascular medicine, including hypertension detection, risk stratification, and outcome prediction [6]. AI-driven approaches leveraging electronic health records (EHRs), wearable devices, ambulatory BP monitoring, and imaging data have demonstrated promising capabilities in identifying undiagnosed hypertension, predicting adverse cardiovascular events, and modeling BP trajectories [7,8]. Nevertheless, most published models focus primarily on algorithmic accuracy rather than implementation within clinical workflows or measurable improvements in patient outcomes.

A critical gap therefore persists between predictive modeling and clinical impact. While AI systems can identify high-risk patients or forecast disease progression, their integration into quality improvement (QI) frameworks capable of reducing therapeutic inertia, improving guideline adherence, and preventing end-organ damage remains insufficiently explored [9,10]. Given that hypertension represents a chronic, system-level condition requiring longitudinal management, AI applications must move beyond prediction toward structured, feedback-driven care optimization. Moreover, hypertension is not merely a disorder of elevated BP but a progressive systemic disease contributing to structural and functional injury across multiple organ systems. Chronic uncontrolled hypertension accelerates cardiac remodeling and heart failure development, promotes vascular remodeling and aortic pathology, and increases the risk of renal and cerebrovascular disease [3,11]. Preventing these downstream complications requires early identification of high-risk phenotypes, timely treatment escalation, and coordinated care pathways, areas in which AI may offer transformative potential.

In this context, the present review aims to critically evaluate artificial intelligence–driven hypertension management through the lens of quality improvement and prevention of end-organ damage. Rather than focusing exclusively on algorithmic performance, we examine how AI can be embedded within primary care–centered clinical workflows, integrated into continuous QI frameworks, and aligned with outcome-oriented hypertension management strategies. By shifting the emphasis from prediction to implementation and organ protection, we seek to outline a pragmatic pathway for translating AI innovation into measurable improvements in cardiovascular health.

## 2. Methods

This narrative review was conducted to synthesize current evidence on artificial intelligence-driven hypertension management, with emphasis on quality improvement and prevention of end-organ damage. A structured literature search was performed in PubMed/MEDLINE and Scopus databases, covering publications from January 2010 through December 2025. The starting year of 2010 was selected to capture the contemporary era of machine learning applications in cardiovascular medicine, coinciding with the rapid expansion of electronic health record adoption and early AI-driven risk modeling in hypertension research. Search terms included combinations of “hypertension”, “artificial intelligence”, “machine learning”, “deep learning,” “natural language processing,” “quality improvement,” “primary care,” “cardiovascular risk,” “heart failure,” “aortic disease”, “chronic kidney disease”, and “stroke”, using Boolean operators (“AND”, “OR”) as appropriate. No language restrictions were applied; however, given the scope and focus of this review, all included studies were published in English. Gray literature, including regulatory guidance documents from the U.S. Food and Drug Administration and the European Medicines Agency, was additionally consulted for the implementation and regulatory sections of this review.

The initial search yielded approximately 1200 records across both databases. Following removal of duplicates, titles and abstracts of approximately 900 records were screened for relevance. Full-text review was performed for approximately 180 articles deemed potentially eligible based on title and abstract screening. Of these, 19 articles were selected for inclusion from the database search. An additional 21 records were identified through manual screening of reference lists of selected articles, yielding a total of 40 peer-reviewed articles included in the narrative synthesis. Original research articles, randomized clinical trials, observational cohort studies, implementation studies, and high-impact narrative or state-of-the-art reviews were considered eligible. Preference was given to peer-reviewed publications in high-impact cardiovascular, nephrology, neurology, and general medical journals. Studies focusing exclusively on technical algorithm development without clinical applicability were deprioritized unless directly relevant to cardiovascular risk modeling or implementation frameworks. Studies were prioritized for inclusion based on: (1) clinical relevance to hypertension detection, risk stratification, or end-organ damage prevention; (2) methodological rigor, including external validation where applicable; (3) relevance to quality improvement or implementation science; and (4) recency, with particular emphasis on publications from 2018 onward.

**Figure 1:**
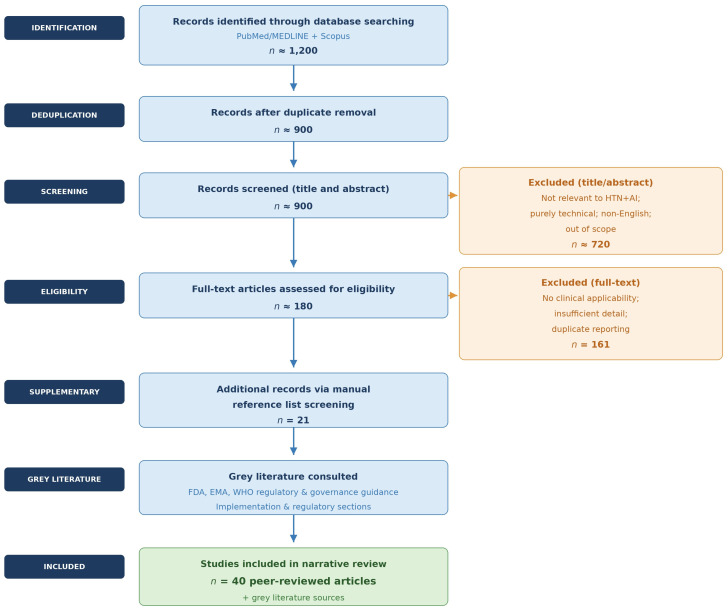
Literature selection flow diagram for the present narrative review. Abbreviations: n = number; HTN = hypertension; AI = artificial intelligence; FDA = Food and Drug Administration; EMA = European Medicines Agency; WHO = World Health Organization.

Given the conceptual and implementation-oriented objectives of this review, formal risk-of-bias assessment and quantitative synthesis were not performed. The literature selection process across all screening stages is summarized in Figure 1. The decision not to conduct a meta-analysis was based on the following prespecified considerations: (1) substantial clinical heterogeneity across included studies, reflecting diverse hypertension phenotypes, comorbidity profiles, and care settings; (2) methodological heterogeneity in AI approaches, spanning supervised machine learning, deep learning, natural language processing, and hybrid architectures with incompatible feature sets and training procedures; (3) heterogeneity in outcome definitions, with studies variously reporting algorithmic discrimination metrics (AUC, C-statistic), clinical process measures (blood pressure control rates), and intermediate outcomes (organ damage biomarkers) that are not amenable to pooled statistical synthesis; and (4) inconsistent reporting of external validation and learning dynamics across studies, which would render pooled performance estimates uninterpretable without standardized quality assessment.

## 3. Overview of Artificial Intelligence Methods Relevant to Hypertension

Artificial intelligence (AI) encompasses a broad range of computational approaches designed to extract patterns, generate predictions, and support decision-making from complex datasets. In the context of hypertension management, AI applications predominantly rely on machine learning (ML), deep learning (DL), natural language processing (NLP), and computer vision techniques applied to EHRs, physiologic signals, and imaging data [12].

Machine learning methods, including supervised and unsupervised algorithms, have been widely used for hypertension risk prediction and phenotyping. Supervised learning approaches—such as logistic regression with regularization, random forests, gradient boosting machines, and support vector machines—are commonly employed to predict incident hypertension, uncontrolled blood pressure, or cardiovascular events using structured EHR variables [13]. Unsupervised learning techniques, including clustering algorithms, enable identification of distinct hypertension phenotypes based on blood pressure variability, comorbidity patterns, and longitudinal trajectories [14].

Deep learning represents a subset of machine learning characterized by multilayer neural networks capable of processing high-dimensional data. In cardiovascular medicine, DL approaches have demonstrated strong performance in imaging interpretation, waveform analysis, and multimodal data integration [15]. Convolutional neural networks (CNNs) have been applied to imaging modalities and physiologic signals, while recurrent neural networks (RNNs) and transformer-based architectures facilitate temporal modeling of longitudinal blood pressure measurements and clinical events.

Natural language processing enables extraction of clinically relevant information from unstructured data sources, such as physician notes, discharge summaries, and referral documentation [16]. NLP-based systems can identify undocumented hypertension diagnoses, capture medication changes, and detect barriers to treatment intensification that are not reflected in structured data fields. This capability is particularly relevant for quality improvement initiatives, where understanding workflow and documentation patterns is essential.

Computer vision techniques extend AI applications to imaging-based assessment of cardiovascular structure and vascular pathology. Automated analysis of echocardiography, computed tomography (CT), and magnetic resonance imaging (MRI) data enables quantification of cardiac remodeling, vascular dimensions, and aortic morphology with increasing precision [17]. Integration of imaging-derived features with longitudinal blood pressure data and EHR variables offers the potential for multimodal risk stratification beyond traditional clinic-based measurements.

Despite their methodological diversity, these AI approaches share common prerequisites for successful clinical translation: high-quality data, external validation, interpretability, and integration into clinical workflows. As the field evolves, the emphasis is shifting from isolated algorithm development toward deployable systems capable of supporting real-time clinical decision-making and continuous quality improvement.

### Comparative Appraisal of AI Paradigms in Hypertension Management

While each AI methodology offers distinct capabilities, a critical appraisal of their comparative strengths and limitations is essential for contextualizing their roles in hypertension management and avoiding uncritical enthusiasm for any single approach. Traditional supervised machine learning methods (including logistic regression with regularization, random forests, and gradient boosting machines) offer the advantage of relative interpretability, computational efficiency, and strong performance on structured tabular data derived from electronic health records [13]. These methods are well-suited to hypertension risk prediction and phenotyping tasks where input features are pre-specified and datasets are moderately sized. Their principal limitation is an inability to exploit unstructured or high-dimensional data, such as imaging, waveforms, or free-text clinical notes, without extensive feature engineering.

Deep learning architectures, by contrast, excel at extracting latent representations from high-dimensional inputs, enabling strong performance in imaging analysis, ECG interpretation, and temporal modeling of longitudinal blood pressure trajectories [15,18]. Nonetheless, deep learning models typically require substantially larger training datasets, are more computationally intensive, and are inherently less interpretable than traditional ML approaches. This constitutes a critical limitation in clinical settings where transparency and auditability are regulatory and ethical requirements [19]. Furthermore, deep learning models are particularly susceptible to performance degradation when deployed in settings with different data distributions than the training environment, amplifying generalizability concerns.

Natural language processing occupies a complementary niche, enabling extraction of clinically relevant information from unstructured documentation that structured EHR fields fail to capture, including undocumented hypertension diagnoses, medication side effects, and patient-reported barriers to adherence [16]. NLP approaches are less frequently evaluated in the hypertension literature relative to structured ML methods, and their performance is highly sensitive to variability in clinical documentation practices across institutions. Large language models (LLMs) and transformer-based architectures represent an emerging frontier, with potential applications in clinical summarization, patient communication, and guideline-adherent decision support. However, their application to hypertension management remains largely theoretical at present, and critical concerns regarding hallucination, calibration, and regulatory oversight remain unresolved. Across all paradigms, a consistent finding is that no single AI methodology dominates across all hypertension management tasks. The optimal approach is likely task-specific: traditional ML for structured risk prediction, deep learning for imaging and waveform analysis, NLP for documentation mining, and hybrid architectures for multimodal integration. This heterogeneity underscores the importance of matching algorithmic choice to clinical task requirements rather than defaulting to methodological novelty.

## 4. AI Applications in Hypertension Detection and Risk Stratification

### 4.1. Diagnosis and Screening

Despite the high prevalence of hypertension, a substantial proportion of individuals remain undiagnosed or inadequately monitored, particularly in primary care settings [20]. Electronic health record–based machine learning models have been developed to identify patients with probable undiagnosed hypertension by analyzing repeated elevated blood pressure measurements, visit patterns, comorbidities, and medication histories [21]. Such algorithms can systematically flag high-risk individuals for confirmatory evaluation, thereby addressing gaps in detection that traditional rule-based approaches may overlook.

Beyond static classification, predictive models have also been constructed to estimate the risk of incident hypertension among normotensive individuals. Leveraging longitudinal EHR data, laboratory values, and demographic features, machine learning systems have demonstrated improved discrimination compared with conventional regression-based risk scores [8]. These approaches enable proactive identification of high-risk patients who may benefit from early lifestyle intervention or closer surveillance. For example, Hwang et al. reported AUROCs of 0.78–0.82 for ML-based incident hypertension prediction across two independent national cohorts in South Korea and Japan, outperforming conventional logistic regression benchmarks [8].

Ambulatory and home blood pressure monitoring have further expanded the data available for AI-driven analysis. Wearable devices and connected blood pressure cuffs generate high-frequency measurements that allow assessment of blood pressure variability, circadian patterns, and masked or white-coat hypertension [22]. Machine learning models trained on these datasets can detect abnormal patterns and predict future cardiovascular risk with increasing precision [23]. Importantly, integration of remote monitoring platforms into clinical workflows provides an opportunity to extend hypertension screening beyond episodic clinic encounters. Notably, a small but growing number of AI-assisted hypertension screening tools have progressed beyond the research stage toward real-world clinical deployment. The AHA’s Target: BP initiative and related quality improvement programs have incorporated algorithmic flagging of uncontrolled hypertension within EHR platforms in primary care networks across the United States [24]. Similarly, AI-enhanced ambulatory blood pressure analysis has been integrated into commercial remote monitoring platforms, though prospective outcome data from these deployments remain limited. The distinction between research-stage models, pilot implementations, and systems with demonstrated real-world effectiveness is critical for contextualizing the current evidence base and setting realistic expectations for near-term clinical impact.

### 4.2. Risk Stratification Beyond Absolute Blood Pressure

Traditional hypertension management has largely relied on single or averaged clinic blood pressure values. However, accumulating evidence suggests that blood pressure variability, longitudinal trajectories, and cumulative exposure may better reflect cardiovascular risk [25]. Machine learning approaches are particularly suited to modeling complex temporal patterns and high-dimensional interactions among clinical variables. These models often outperform traditional risk scores in discrimination. For instance, Ambale-Venkatesh et al. demonstrated improved discrimination over conventional Framingham-based risk scores [13], although calibration and external validation remain variable.

Supervised learning models have been used to predict adverse cardiovascular outcomes in hypertensive populations, incorporating demographic characteristics, comorbidities, laboratory values, and medication adherence data [13]. These models often outperform traditional risk scores in discrimination, although calibration and external validation remain variable. In parallel, unsupervised clustering techniques have enabled identification of distinct phenotypic subgroups within hypertensive cohorts, characterized by differing comorbidity burdens, response to therapy, and risk profiles [26]. Such phenomapping approaches may facilitate more individualized treatment strategies.

Multimodal models integrating structured EHR data, imaging features, and physiologic signals represent a further evolution in risk stratification [27]. For example, AI-assisted imaging analysis can quantify left ventricular remodeling or vascular changes associated with chronic hypertension, augmenting clinical risk assessment beyond blood pressure values alone [17]. These integrative strategies offer the potential to identify patients at heightened risk for progression to heart failure, aortic pathology, or other end-organ complications before overt clinical deterioration. The breadth of current artificial intelligence applications in hypertension, spanning detection, risk modeling, and organ-specific assessment, is summarized in Table 1. Beyond modeling chronic risk trajectories, machine learning techniques may also facilitate pattern recognition of hypertensive crises and malignant hypertension events. By analyzing longitudinal blood pressure variability, abrupt elevations, medication adherence patterns, and comorbidity profiles, AI systems could potentially identify patients at imminent risk for acute hypertensive complications. Early recognition of such high-risk patterns may support proactive treatment intensification or expedited evaluation, particularly in patients with poorly controlled or labile hypertension. However, prospective validation of such predictive approaches remains limited.

### 4.3. Limitations and Generalizability of Current AI Models

While AI-driven hypertension models show promising predictive performance, several limitations constrain their current clinical applicability. Many models are developed within single health systems using retrospective datasets, raising concerns regarding transportability across diverse populations and practice environments [30]. Beyond external validation, a related and frequently overlooked methodological requirement is the reporting of learning dynamics (including learning curves and overfitting checks) which are necessary to determine whether high reported accuracy reflects true model generalizability rather than overfitting to training data [30]. Among the studies reviewed, systematic reporting of learning dynamics is the exception rather than the rule, representing a critical gap in methodological transparency. Readers and clinicians should therefore interpret reported performance metrics with caution when learning dynamics and independent external validation are absent. External validation is inconsistently performed, and performance may degrade when applied to new settings with differing demographic or socioeconomic characteristics.

Algorithmic bias is another critical concern. Models trained on EHR data may inadvertently reflect existing disparities in access to care, documentation practices, or treatment patterns, thereby amplifying inequities if deployed without careful evaluation [31]. Transparency, interpretability, and clinician trust remain central to successful implementation, particularly in primary care settings where time constraints and alert fatigue are prevalent [19].

Collectively, these considerations underscore that predictive accuracy alone is insufficient. For AI systems to meaningfully improve hypertension outcomes, they must be externally validated, embedded within clinical workflows, and aligned with quality improvement objectives.

### 4.4. Cross-Study Synthesis: Points of Consensus, Divergence, and Uncertainty

Several consistent patterns emerge across the AI-hypertension literature, alongside important areas of divergence that warrant critical appraisal. Below are the most important:

Areas of consensus: Studies broadly agree that machine learning models outperform conventional regression-based risk scores in discrimination for incident hypertension prediction and cardiovascular event risk stratification [7,8,13,32]. There is also consistent agreement that imaging-derived features, electrocardiographic biomarkers, and longitudinal blood pressure variability metrics add predictive value beyond clinic-based measurements [17,18,25]. The importance of external validation as a prerequisite for clinical deployment is acknowledged across virtually all methodological frameworks [30,33].

Areas of divergence: Despite general agreement on discriminative superiority, reported performance metrics vary substantially across studies, thus reflecting differences in cohort demographics, outcome definitions, follow-up duration, and feature sets. Models developed in single health systems frequently fail to maintain performance when applied to external populations [30,33], and calibration, the agreement between predicted and observed risk, is inconsistently reported. Furthermore, studies differ markedly in their inclusion of learning dynamics: few systematically report learning curves or overfitting checks, making it difficult to determine whether high reported accuracy reflects true generalizability or model overfitting to training data.

Potential reasons for divergence: Heterogeneity in study populations, variable data quality across electronic health record systems, differences in blood pressure measurement protocols, and inconsistent handling of missing data collectively contribute to performance variability. Additionally, the choice of comparator (whether a simple logistic regression or an established risk score such as the Framingham or ACC/AHA models) substantially influences the magnitude of reported ML superiority.

Remaining uncertainties: A critical and unresolved question is whether improvements in algorithmic discrimination translate into measurable improvements in clinical outcomes. The gap between model performance metrics and patient-level benefit remains largely unquantified, and prospective pragmatic trials directly linking AI-driven hypertension interventions to reductions in cardiovascular events are still lacking.

## 5. AI as a Tool for Quality Improvement in Hypertension Care

### 5.1. Therapeutic Inertia and Guideline Adherence

Suboptimal blood pressure control is frequently driven not only by patient-related factors but also by therapeutic inertia, defined as the failure to initiate or intensify therapy when indicated [34]. Therapeutic inertia has been consistently associated with persistent uncontrolled hypertension and increased cardiovascular risk [35]. Despite clear treatment thresholds and intensification recommendations in contemporary hypertension guidelines [3], delays in medication adjustment remain common in routine practice. A clinically important distinction must be drawn between three categories of AI tools currently described in the hypertension literature: (1) research-stage models developed and tested within single health systems using retrospective data, which represent the majority of published studies; (2) prospectively validated tools that have demonstrated performance in independent cohorts or controlled trial settings, such as the HOME BP digital intervention evaluated by McManus et al. [23]; and (3) clinically deployed systems integrated into routine care workflows, for which real-world effectiveness data are beginning to emerge but remain sparse. Conflating these categories risks overstating the current clinical readiness of AI-driven hypertension management and undervaluing the substantial implementation work that remains.

Clinical decision support systems (CDSS), increasingly augmented by machine learning techniques, have been proposed as tools to mitigate therapeutic inertia by providing real-time, patient-specific treatment recommendations [36]. In primary care environments, CDSS interventions integrated within electronic health records have demonstrated modest but meaningful improvements in blood pressure control when aligned with workflow and provider engagement strategies [37]. These findings suggest that algorithmic support alone is insufficient; effectiveness depends on thoughtful implementation within clinical practice. Of note, AI-enhanced systems may extend beyond rule-based alerts by incorporating longitudinal patient data, medication adherence patterns, and risk stratification models to recommend personalized treatment escalation pathways. However, successful deployment requires alignment with guideline-based targets and clinician trust to avoid alert fatigue and unintended workflow disruption.

### 5.2. AI Embedded in Quality Improvement Frameworks

Quality improvement frameworks such as the Model for Improvement and Plan–Do–Study–Act (PDSA) cycles provide structured approaches to translating evidence into measurable practice change [37,38]. When embedded within such frameworks, AI tools can function as components of a continuous feedback loop: identifying care gaps, generating targeted interventions, and enabling outcome monitoring.

Audit and feedback interventions, a core QI strategy, have demonstrated small-to-moderate improvements in professional practice across diverse healthcare settings [38,39]. AI systems can enhance audit and feedback processes by automating performance measurement, identifying high-risk patient subgroups, and delivering tailored performance dashboards to clinicians. In this context, AI does not replace established QI methodologies but augments their scalability and precision.

Implementation science frameworks such as the Consolidated Framework for Implementation Research (CFIR) offer structured approaches to understanding contextual factors that influence adoption of digital health tools [39,40]. Integration of AI-driven hypertension programs within primary care settings requires attention to workflow compatibility, organizational readiness, leadership engagement, and measurement of real-world effectiveness. Without such structured implementation strategies, predictive models risk remaining confined to research environments. The principal domains in which AI can be operationalized within structured hypertension quality improvement programs are outlined in Table 2.

### 5.3. AI-Driven Versus Non-AI Quality Improvement Interventions: A Critical Comparison

Before AI can be positioned as a transformative force in hypertension quality improvement, it must be critically compared against the established non-AI QI interventions with which it would compete or integrate in real-world clinical systems. Several non-AI strategies have demonstrated robust, replicated evidence for improving hypertension control and deserve explicit acknowledgment as the benchmark against which AI-driven approaches must be evaluated.

Pharmacist-led medication management and collaborative drug therapy management programs have consistently demonstrated reductions in systolic blood pressure of 5–10 mmHg in randomized trials, with strong evidence for improving medication adherence and guideline concordance [36]. Team-based care models integrating nurse practitioners, clinical pharmacists, and care coordinators within primary care have similarly shown meaningful improvements in blood pressure control rates in large pragmatic trials. Audit and feedback interventions, a cornerstone of traditional QI, have demonstrated small-to-moderate improvements in clinical performance across diverse settings [38], and the Plan-Do-Study-Act cycle has been the structural backbone of most successful hypertension QI programs [37]. Against this evidence base, the comparative value of AI-driven QI interventions remains uncertain. Current AI-based clinical decision support systems have demonstrated modest improvements in blood pressure control in select settings [36], but head-to-head comparisons against pharmacist-led or team-based care models are largely absent from the literature. Crucially, non-AI interventions benefit from decades of implementation experience, established workflow integration, and a well-characterized evidence base—advantages that nascent AI systems have not yet accumulated. The most defensible positioning for AI-driven hypertension QI, therefore, is not as a replacement for established non-AI strategies but as a precision augmentation layer: identifying patients most likely to benefit from intensified pharmacist involvement, flagging care gaps before team-based escalation, and enabling population-level monitoring that individual clinicians cannot perform manually. This hybrid model (combining the scalability of AI with the proven effectiveness of team-based and audit-driven QI) represents the most evidence-aligned pathway for AI integration into hypertension management programs. Until prospective comparative data are available, the incremental contribution of AI over and above established QI strategies cannot be assumed and should be explicitly quantified in future trial designs.

### 5.4. Measuring What Matters: From Blood Pressure to Clinical Outcomes

While blood pressure reduction remains a central therapeutic goal, its use as a sole process metric may not fully capture improvements in long-term cardiovascular outcomes. Landmark randomized trials have demonstrated that intensive blood pressure control reduces major cardiovascular events and mortality [40,41]. However, translating trial-level targets into population-level improvements requires robust systems for performance measurement and outcome linkage.

Professional societies have developed standardized hypertension performance and quality measures to assess clinical care processes and outcomes [24]. AI-driven analytics can enhance these efforts by identifying patterns of suboptimal control, monitoring time-to-intensification, and correlating care processes with downstream events such as heart failure hospitalization or stroke.

Accordingly, AI-driven hypertension programs should prioritize outcome-oriented evaluation frameworks that move beyond isolated BP readings to incorporate cardiovascular event reduction, prevention of end-organ damage, and equity metrics. Aligning predictive analytics with measurable clinical outcomes is essential to justify widespread implementation.

### 5.5. The Evidence Gap: From Algorithmic Performance to Clinical Outcomes

A fundamental tension running through the AI-hypertension literature is the disconnect between algorithmic performance metrics and demonstrated improvements in patient outcomes. The majority of published studies report discrimination metrics—most commonly the area under the receiver operating characteristic curve (AUC or C-statistic) —as their primary measure of model success. While discrimination is a necessary prerequisite for clinical utility, it is insufficient evidence of clinical benefit. A model with an AUC of 0.85 may identify high-risk patients accurately yet fail to improve outcomes if its predictions are not acted upon, are not actionable within existing workflows, or target populations in whom effective interventions are unavailable. To date, evidence that AI-driven hypertension interventions produce measurable improvements in clinical outcomes, including blood pressure control rates, cardiovascular event reduction, or prevention of end-organ damage, remains limited. The HOME BP randomized trial by McManus et al. represents one of the few prospective evaluations of a digitally supported hypertension management intervention, demonstrating meaningful reductions in systolic blood pressure compared with usual care [23]. Similarly, clinical decision support systems evaluated in randomized trials have shown modest improvements in blood pressure control when integrated thoughtfully within primary care workflows [36]. However, longer-term outcome data linking these interventions to reductions in heart failure hospitalization, stroke incidence, renal decline, or aortic events are largely absent from the current literature.

This evidence gap reflects a broader challenge in implementation science: the distance between algorithmic development and clinical impact is rarely bridged by a single study. Translating predictive models into outcome improvements requires prospective implementation studies designed to evaluate not only whether AI tools perform as intended, but whether their deployment within real-world clinical systems produces measurable patient benefit. Pragmatic clinical trials (conducted within routine care settings, enrolling diverse populations, and measuring outcomes beyond blood pressure) are therefore an essential next step for the field. Until such evidence exists, AI-driven hypertension management should be positioned as a promising adjunct to, rather than a replacement for, established quality improvement strategies with demonstrated outcome benefit.

### 5.6. Primary Care–Centered AI for Hypertension Quality Improvement

Because the majority of hypertension diagnosis and longitudinal management occurs in primary care, AI-driven quality improvement efforts must be designed with this setting as the central implementation environment [20]. EHR-based algorithms have demonstrated the ability to identify patients with probable undiagnosed or uncontrolled hypertension within primary care populations [21]. When coupled with structured follow-up protocols, such tools can facilitate earlier confirmation of diagnosis and timely treatment intensification.

Large-scale public health initiatives, such as the Million Hearts campaign, have underscored the importance of population-level blood pressure control strategies within primary care systems [42]. AI-enhanced population health dashboards may further support these efforts by stratifying patients according to risk, identifying care gaps, and guiding referral pathways for specialty evaluation when appropriate.

**Figure 2:**
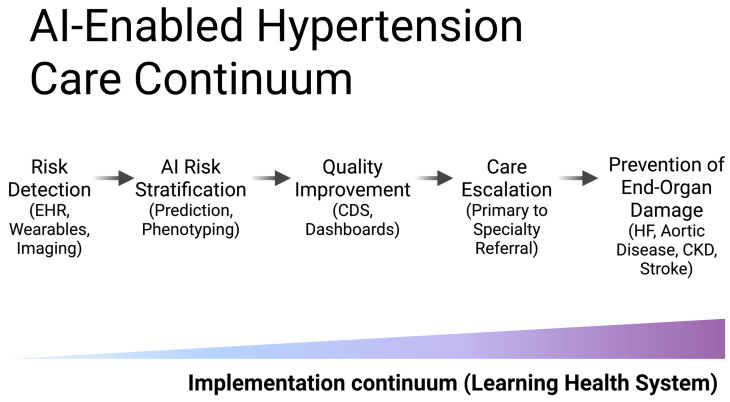
Conceptual framework of artificial intelligence–driven hypertension management. Artificial intelligence integrates multimodal data sources, including electronic health records, wearable devices, and imaging, to enable risk detection and stratification in patients with or at risk for hypertension. When embedded within structured quality improvement frameworks in primary care settings, AI supports therapeutic intensification, care escalation, and referral pathways. Continuous performance monitoring and outcome evaluation facilitate prevention of hypertension-related end-organ damage (including heart failure, aortic disease, chronic kidney disease, and stroke) within a learning health system model characterized by iterative feedback and system-level improvement. The stages depicted in this framework are supported by empirical evidence at varying levels of maturity. Detection and screening algorithms have demonstrated AUCs exceeding 0.80 in large EHR-based cohorts [7,8], while risk stratification models integrating multimodal data have shown improved discrimination over conventional scores across multi-ethnic populations [13,32]. Clinical decision support interventions embedded within primary care workflows have produced modest but measurable improvements in blood pressure control in randomized trials [23,36]. By contrast, the outcome monitoring and recalibration stages remain primarily aspirational, with prospective evidence linking AI-driven interventions to reductions in end-organ damage still limited. Created in BioRender. Magouliotis, D. (2026) https://BioRender.com/3dzc85i (accessed on 23 March 2026). Abbreviations: EHR = electronic health record; CDS = clinical decision support; HF = heart failure; CKD = chronic kidney disease; AI = artificial intelligence.

However, deployment in primary care requires careful attention to clinician workload, documentation burden, and alert fatigue [19]. AI systems that integrate seamlessly into existing workflows and provide concise, actionable recommendations are more likely to achieve sustained adoption. Ultimately, primary care–centered implementation represents the critical bridge between predictive modeling and meaningful reductions in hypertension-related end-organ damage. The proposed conceptual framework for artificial intelligence–driven hypertension management, integrating risk detection, quality improvement, and prevention of end-organ damage within a continuous learning health system model, is illustrated in Figure 2.

## 6. AI and Prevention of Hypertension-Related End-Organ Damage

Hypertension is not merely a hemodynamic abnormality but a progressive systemic disorder that drives structural and functional injury across multiple organ systems. Chronic exposure to elevated blood pressure accelerates cardiac remodeling, promotes vascular wall degeneration, impairs renal function, and increases cerebrovascular risk [42]. Accordingly, prevention of end-organ damage represents the ultimate objective of hypertension management. Artificial intelligence–driven approaches may facilitate earlier identification of patients at risk for irreversible structural disease and enable targeted intervention strategies.

### 6.1. Cardiac Remodeling and Heart Failure

Long-standing hypertension contributes to left ventricular hypertrophy, myocardial fibrosis, and diastolic dysfunction, ultimately increasing the risk of heart failure—particularly heart failure with preserved ejection fraction (HFpEF) [43]. Traditional risk stratification strategies rely primarily on clinic-based blood pressure measurements and clinical comorbidities; however, these approaches may inadequately capture subclinical remodeling processes.

AI-driven imaging analysis has demonstrated the capacity to quantify cardiac structure and function with high precision. Deep learning models applied to echocardiographic data can automatically assess left ventricular ejection fraction and structural parameters, facilitating scalable detection of remodeling patterns associated with hypertensive heart disease [18]. Beyond imaging, machine learning-based phenomapping approaches have identified distinct heart failure phenotypes, many driven by hypertension as the primary substrate, with differing comorbidity profiles and outcomes, illustrating how unsupervised clustering can refine hypertension-related risk stratification beyond conventional categories [26].

Integration of longitudinal blood pressure data, EHR variables, and imaging-derived features may enable earlier prediction of progression from asymptomatic hypertensive heart disease to overt heart failure. Such multimodal models offer the potential to identify high-risk patients who may benefit from intensified blood pressure control, neurohormonal modulation, or closer surveillance before symptomatic deterioration occurs. Hypertension also contributes to atrial structural remodeling and increased susceptibility to atrial fibrillation (AF), which in turn amplifies stroke risk. AI-driven electrocardiographic analysis has demonstrated the ability to detect latent or paroxysmal AF and to identify electrical signatures associated with atrial remodeling even during sinus rhythm. Integration of AI-based AF detection with hypertension management strategies may therefore enhance cerebrovascular risk stratification and inform more aggressive blood pressure control or rhythm monitoring strategies in selected high-risk individuals.

Beyond cardiac and cerebrovascular manifestations, hypertensive retinopathy represents an additional and underappreciated end-organ target accessible to AI-driven assessment. Deep learning algorithms applied to retinal fundus imaging have demonstrated strong discriminative performance in detecting hypertensive retinal changes, including arteriolar narrowing, arteriovenous nicking, and hemorrhages, and have shown potential for non-invasive cardiovascular risk stratification from retinal vascular morphology alone. A deep learning system for the assessment of cardiovascular disease risk via retinal vessel calibre measurement, developed by Cheung et al. using data from large population-based Asian cohorts, achieved AUCs of up to 0.85 for hypertension detection and demonstrated improved cardiovascular risk stratification beyond traditional risk factors [44]. Furthermore, Poplin et al. demonstrated that deep learning models trained on retinal fundus photographs from 284,335 patients could predict systolic blood pressure with a mean absolute error of approximately 11 mmHg and identify patients at risk of major adverse cardiac events with an AUC of approximately 0.70 [45]. While this application remains primarily at the research stage, it illustrates the expanding scope of imaging-based AI in capturing the systemic vascular consequences of chronic hypertension.

### 6.2. Vascular Remodeling and Aortic Disease

Hypertension is a well-recognized risk factor for thoracic and abdominal aortic aneurysm formation and progression, as well as for aortic dissection [46]. Elevated mechanical stress contributes to medial degeneration, elastin fragmentation, and progressive vascular dilation. Despite established associations between hypertension and aortic pathology, risk stratification for aneurysm growth and rupture remains largely diameter-based and reactive.

Artificial intelligence has increasingly been applied to cardiovascular imaging to improve detection and characterization of vascular disease. Deep learning algorithms can automate measurement of aortic dimensions and extract imaging-derived features associated with structural instability [17]. Machine learning models integrating clinical variables and imaging characteristics have shown promise in predicting aneurysm expansion and adverse aortic events, although most studies remain retrospective and require prospective validation [28].

In hypertensive populations, combining cumulative blood pressure burden, variability metrics, and imaging-derived vascular parameters may improve identification of patients at heightened risk for progressive aortic remodeling. Such approaches could support personalized surveillance intervals, earlier specialist referral, and optimized antihypertensive therapy aimed at reducing wall stress. Importantly, embedding these predictive tools within structured quality improvement pathways may facilitate earlier intervention before catastrophic events occur.

### 6.3. Renal and Cerebrovascular Injury

Hypertension is a leading contributor to chronic kidney disease (CKD) and ischemic stroke [42]. Renal microvascular damage, glomerulosclerosis, and progressive decline in estimated glomerular filtration rate are closely linked to long-standing blood pressure elevation. Machine learning models using EHR data have demonstrated improved prediction of incident CKD and progression compared with conventional risk equations [47]. Early identification of hypertensive patients at high risk for renal decline may allow intensification of renin–angiotensin system blockade, tighter blood pressure control, and nephrology referral. Similarly, AI-based risk models incorporating blood pressure variability, atrial fibrillation status, and imaging markers have been developed to predict stroke risk beyond traditional scoring systems [29]. In this context, AI may serve as a decision-support tool to guide more aggressive hypertension management in individuals at elevated cerebrovascular risk.

Collectively, these applications illustrate how AI-driven hypertension management can shift the focus from reactive treatment of established organ damage toward proactive identification of structural vulnerability. However, predictive capability must be coupled with structured implementation strategies to translate risk identification into improved clinical outcomes.

### 6.4. Early Identification of Acute Cardiovascular Events 

Beyond long-term structural remodeling, uncontrolled hypertension is a primary driver of acute life-threatening events, including hypertension-related ischemic stroke, hypertensive emergency with myocardial infarction, and acute aortic dissection, each representing a failure of chronic blood pressure management [40,42,46]. Artificial intelligence systems applied to EHR data, imaging, and physiologic monitoring have demonstrated potential for early risk detection and triage support. Predictive analytics capable of identifying deteriorating hemodynamic patterns, abrupt blood pressure surges, or evolving imaging abnormalities may enable earlier intervention in emergency or outpatient settings. Although most existing models focus on diagnostic augmentation rather than prevention, integration of such systems within hypertension management programs could support timely escalation of care and reduce morbidity associated with acute hypertensive complications.

### 6.5. A Mechanistic Taxonomy: Linking Data Modality to Organ-Specific Damage Pathways

A critical gap in the current AI-hypertension literature is the absence of a structured framework connecting the type of data that AI systems consume to the specific organ-damage pathways they are designed to detect or prevent. Such a taxonomy is mechanistically important: different end-organ complications of hypertension arise through distinct pathophysiological mechanisms, and the data modalities most informative for predicting each complication reflect these underlying biological processes. Hypertensive heart disease, encompassing left ventricular hypertrophy, diastolic dysfunction, and progression to heart failure, is mechanistically driven by chronic pressure overload, neurohormonal activation, and myocardial fibrosis [43]. AI systems targeting this pathway are therefore most informative when trained on electrocardiographic data capturing electrical remodeling signatures, echocardiographic imaging quantifying chamber dimensions and filling pressures, and longitudinal blood pressure variability metrics reflecting cumulative hemodynamic stress [18]. The biological signal linking these data modalities to the damage pathway is well-established, supporting the mechanistic validity of AI models in this domain.

Aortic remodeling and aneurysm progression are driven by medial degeneration, elastin fragmentation, and wall stress—processes that manifest primarily in structural imaging features rather than in laboratory or EHR variables alone [46]. AI systems targeting aortic pathology therefore derive their greatest predictive value from CT- and MRI-derived morphological features, including aortic diameter trajectories, wall thickness, and biomechanical indices, augmented by blood pressure burden metrics as a surrogate for cumulative wall stress [28]. The mechanistic link between imaging-derived structural parameters and aortic damage pathways justifies the imaging-centric design of current AI aortic risk models. Hypertensive nephropathy—characterized by glomerulosclerosis, tubulointerstitial fibrosis, and progressive decline in glomerular filtration—is most sensitively captured through longitudinal laboratory trajectories, particularly serial estimated glomerular filtration rate and albuminuria measurements, which reflect cumulative microvascular injury [47]. AI systems integrating these laboratory biomarkers with blood pressure control metrics and renin-angiotensin system medication histories are mechanistically well-positioned to predict renal decline trajectories.

Cerebrovascular injury encompasses both large-vessel ischemic stroke—driven by atherosclerosis, embolism, and hemodynamic compromise—and small-vessel disease reflecting chronic microvascular injury from sustained hypertension. The mechanistic heterogeneity of cerebrovascular events implies that optimal AI prediction requires multimodal integration: blood pressure variability metrics, atrial fibrillation detection from ECG or wearable data, imaging-derived markers of white matter hyperintensity burden, and carotid ultrasonography features [29]. This mechanistic taxonomy is summarized in Table 3, which maps primary data modalities to their corresponding organ-damage pathways, the biological mechanism linking them, and the current state of AI evidence in each domain. This framework is intended to guide future AI development by ensuring that algorithmic design is anchored in the pathophysiology of hypertensive end-organ injury rather than driven solely by data availability.

## 7. Implementation Challenges and Ethical Considerations

Although artificial intelligence offers substantial promise for improving hypertension management, translation from predictive modeling to routine clinical practice remains complex. Successful implementation requires careful attention to generalizability, bias, workflow integration, regulatory oversight, and clinician trust.

### 7.1. Generalizability and External Validation

Many AI models in cardiovascular medicine are developed using retrospective data from single health systems, raising concerns about transportability across diverse patient populations [33]. Differences in demographics, comorbidity patterns, socioeconomic factors, and healthcare delivery structures may substantially affect model performance. External validation across multiple institutions and prospective evaluation are therefore essential before large-scale deployment. Generalizability must also account for technical interoperability constraints: AI systems developed within a single EHR environment may fail to replicate performance in institutions using different data architectures or FHIR implementation profiles.

Moreover, calibration—the agreement between predicted and observed outcomes—is often underreported in AI studies. Without robust validation, predictive accuracy observed during model development may not translate into real-world effectiveness. For hypertension programs, this limitation is particularly relevant given the chronic and population-level nature of care delivery.

### 7.2. Algorithmic Bias and Equity

AI systems trained on historical healthcare data may inadvertently perpetuate existing disparities. Differential access to care, variations in documentation practices, and structural inequities can be encoded within EHR-derived datasets [48]. If unaddressed, such biases may disproportionately affect marginalized populations, exacerbating rather than mitigating inequities in hypertension outcomes.

Equity-aware evaluation, subgroup performance reporting, and transparent model auditing are therefore essential components of responsible AI deployment. In hypertension management—where disparities in detection, treatment, and control are well documented—equitable implementation must be considered a core objective rather than a secondary concern.

### 7.3. Workflow Integration and Alert Fatigue

Integration into clinical workflows remains one of the greatest barriers to adoption. Clinical decision-support tools that generate excessive or poorly targeted alerts contribute to alert fatigue and reduced clinician responsiveness [19]. AI systems must therefore prioritize concise, actionable recommendations aligned with existing care pathways. Embedding predictive analytics within primary care–centered workflows requires co-design with clinicians, integration into medication refill processes or follow-up scheduling systems, and alignment with quality improvement initiatives. Standalone predictive dashboards without workflow integration are unlikely to achieve sustained clinical impact.

Operationalizing the frameworks depicted in Figure 1 and Figure 2 within existing healthcare systems requires attention to several concrete infrastructure prerequisites. These include: interoperability between AI platforms and EHR systems, ideally through standardized application programming interfaces (APIs) conforming to Fast Healthcare Interoperability Resources (FHIR) standards; reliable access to structured longitudinal data including blood pressure trajectories, laboratory values, and medication histories; mechanisms for real-time or near-real-time data exchange between remote monitoring devices and clinical decision support systems; and institutional governance frameworks capable of overseeing model performance monitoring, recalibration, and equity auditing over time. Without these foundational infrastructure elements, even well-validated predictive models are unlikely to achieve sustained integration into routine hypertension care. Nonetheless, HL7-FHIR implementation varies substantially across electronic health record vendors and healthcare systems, with differences in supported resource profiles, API conformance levels, and data element mappings. This interoperability heterogeneity means that AI tools validated in one EHR environment may not function as intended when deployed across institutions with different technical infrastructures, representing a concrete and underappreciated barrier to generalizability that extends beyond population-level demographic differences.

### 7.4. Regulatory and Governance Considerations

The regulatory landscape for AI-based medical tools continues to evolve. The U.S. Food and Drug Administration (FDA) has proposed frameworks for software as a medical device (SaMD), including adaptive algorithms capable of post-deployment learning [49]. Clear governance structures, post-marketing surveillance, and continuous performance monitoring are necessary to ensure safety and effectiveness over time. Data privacy and cybersecurity considerations further complicate large-scale implementation, particularly when integrating wearable devices and remote blood pressure monitoring platforms. Transparent data governance policies and patient engagement are essential to maintain trust in AI-driven hypertension programs.

Beyond the FDA framework, international regulatory bodies have developed parallel and complementary oversight structures for AI-based medical devices. The European Union’s Medical Device Regulation (EU MDR 2017/745) establishes a risk-based classification system for AI tools in healthcare, with particular emphasis on transparency, human oversight, and post-market surveillance requirements for high-risk applications [50,51]. The forthcoming EU Artificial Intelligence Act further extends these provisions by imposing specific obligations on high-risk AI systems deployed in healthcare settings, including requirements for human oversight, robustness testing, and transparency documentation [52]. Similarly, the UK Medicines and Healthcare products Regulatory Agency (MHRA) has published guidance on software as a medical device [53], and the World Health Organization has issued global guidance on ethics and governance of AI for health [54]. These frameworks share common principles with the FDA approach—including requirements for clinical validation, bias assessment, and ongoing performance monitoring—but differ in their classification thresholds and post-deployment obligations, creating regulatory heterogeneity that developers of globally deployed AI hypertension tools must navigate carefully.

A related and increasingly prominent consideration is explainable AI (XAI)—methods that render the reasoning of complex models interpretable to clinicians and patients. In hypertension management, where clinical decisions carry significant cardiovascular consequences, black-box models that generate predictions without interpretable reasoning are unlikely to achieve sustained clinician adoption [19]. XAI techniques such as SHapley Additive exPlanations (SHAP) values, Local Interpretable Model-agnostic Explanations (LIME), and attention mechanisms in deep learning architectures provide post hoc or intrinsic interpretability that can support clinical trust, facilitate error detection, and enable regulatory auditability. Incorporating XAI requirements into the design and evaluation of AI-driven hypertension tools should therefore be considered a core implementation standard rather than an optional enhancement.

**Figure 3:**
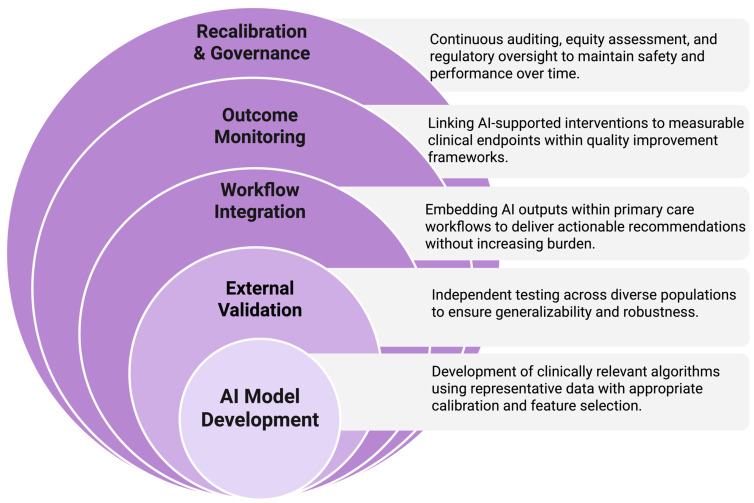
Implementation pathway for artificial intelligence–driven hypertension management in primary care. The framework illustrates the progressive stages required to translate AI models into sustainable clinical practice. Development of clinically relevant algorithms is followed by rigorous external validation to ensure generalizability. Successful deployment requires seamless integration into primary care workflows and alignment with quality improvement frameworks. Continuous outcome monitoring links AI-supported interventions to measurable clinical endpoints. Recalibration and governance, including auditing, equity assessment, and regulatory oversight, are essential to maintain safety, fairness, and performance over time. Each stage of this implementation pathway corresponds to an identifiable evidence gap in the current literature. Algorithm development is the most mature stage, represented by numerous retrospective modeling studies [7,8,13,32]. External validation remains inconsistently performed [30,33]. Workflow integration has been evaluated in a limited number of prospective and randomized studies [23,36,37]. Continuous outcome monitoring and governance frameworks are largely absent from current AI-hypertension research, representing the most critical unmet need for safe clinical deployment. Created in BioRender. Magouliotis, D. (2026) https://BioRender.com/l8a3x09 (accessed on 23 March 2026).

Collectively, these considerations underscore that predictive capability alone does not guarantee improved outcomes. Implementation success depends on rigorous validation, equity-centered design, workflow alignment, and ongoing governance. Addressing these challenges is fundamental to realizing the potential of AI-driven hypertension management. Key barriers to implementation and corresponding mitigation strategies for AI-driven hypertension programs are summarized in Table 4. The sequential pathway required for safe and scalable implementation of AI-driven hypertension programs in primary care settings is illustrated in Figure 3.

## 8. Future Directions: From Algorithms to Systems

The evolution of artificial intelligence in hypertension management is likely to shift from isolated predictive models toward integrated, system-level solutions. Future progress will depend less on incremental gains in algorithmic discrimination and more on the development of scalable, interoperable platforms capable of embedding predictive analytics within routine care delivery.

### 8.1. Multimodal and Longitudinal Risk Modeling

The conceptual frameworks presented in Figure 1 and Figure 2 are intended to provide structural orientation for readers navigating a heterogeneous literature. While these diagrams remain process-oriented by design, the evidence base underlying each stage varies considerably in maturity, as outlined in the figure annotations and the cross-study synthesis presented in Section 4.4. Emerging AI systems increasingly integrate multimodal data streams, including structured EHR variables, imaging-derived features, laboratory biomarkers, wearable-device measurements, and patient-reported outcomes [17]. In hypertension management, such multimodal integration may enable more comprehensive modeling of cumulative blood pressure burden, vascular remodeling, and subclinical organ injury.

Temporal modeling approaches, including recurrent neural networks and transformer-based architectures, offer opportunities to analyze longitudinal trajectories rather than static measurements [44]. Modeling patterns of blood pressure variability, medication adherence, and clinical events over time may improve early identification of patients at risk for heart failure, aortic disease progression, renal decline, or cerebrovascular events.

However, multimodal systems require standardized data architectures and interoperability across healthcare systems. Without robust data governance and harmonization, the potential of longitudinal AI modeling will remain limited.

### 8.2. AI-Driven Hypertension Programs

Rather than functioning solely as background risk calculators, future AI tools may serve as central components of comprehensive hypertension programs. Integration with population health dashboards, automated follow-up reminders, risk-based referral algorithms, and medication titration support systems could enable proactive care management at scale [48]. Learning health system frameworks provide a model for continuous performance improvement, in which data generated during routine care are analyzed, fed back to clinicians, and used to iteratively refine interventions [10]. Embedding AI within such feedback loops may facilitate dynamic adaptation of hypertension management strategies based on real-world outcomes.

### 8.3. Equity-Oriented and Outcome-Driven Implementation

Future AI-driven hypertension care must explicitly prioritize equity and outcome measurement. Given well-documented disparities in hypertension detection and control, algorithm performance should be evaluated across demographic subgroups and linked to measurable reductions in cardiovascular events and end-organ damage [48]. Transparent reporting standards, post-deployment monitoring, and continuous recalibration are essential to ensure sustained benefit. Prospective, pragmatic trials evaluating AI-driven hypertension interventions in primary care settings will be critical to establishing causal impact on clinical outcomes. Demonstrating improvements in heart failure hospitalization rates, prevention of aortic events, renal preservation, and stroke reduction will ultimately determine the clinical value of AI-driven systems.

AI in hypertension management is therefore poised to evolve from predictive modeling toward integrated learning systems that align data analytics with quality improvement and organ protection. The next phase of innovation will depend on multidisciplinary collaboration, rigorous evaluation, and deliberate implementation strategies.

## 9. Conclusions

Artificial intelligence-driven approaches to hypertension management have advanced rapidly, demonstrating promising capabilities in detection, risk stratification, and prediction of cardiovascular events. However, predictive performance alone does not guarantee clinical impact. As hypertension represents a chronic, system-level condition requiring longitudinal care, the true value of AI lies in its ability to support quality improvement, reduce therapeutic inertia, and prevent progressive end-organ damage.

Future progress will depend on embedding AI within primary care-centered workflows, integrating multimodal data sources, ensuring rigorous external validation, and aligning predictive analytics with measurable clinical outcomes. Critically, the field must move beyond reporting algorithmic discrimination metrics as proxies for clinical value. Prospective pragmatic trials measuring the impact of AI-driven hypertension interventions on cardiovascular event rates, end-organ damage prevention, and health equity outcomes are urgently needed to establish the evidence base required for widespread, responsible clinical deployment. Equity-focused evaluation and transparent governance frameworks are essential to avoid perpetuating disparities in hypertension control. Importantly, the success of AI-driven hypertension programs should ultimately be judged not by improvements in algorithmic metrics, but by reductions in heart failure incidence, aortic complications, renal decline, and cerebrovascular events.

By shifting the focus from isolated prediction models to integrated, outcome-oriented systems, artificial intelligence may help transform hypertension care from reactive blood pressure control toward proactive organ protection. Realizing this potential will require multidisciplinary collaboration, pragmatic implementation studies, and sustained commitment to evidence-based quality improvement.

## Figures and Tables

**Table 1:** Comparative Summary of Key Studies on AI Applications in Hypertension and Cardiovascular End-Organ Risk.

Study (Author, Year)	Study Design	Population	AI Methodology	Key Finding (Abstract-Verified)	External Validation	Learning Dynamics Reported
Hwang et al., 2024 [8]	Retrospective cohort	2 national cohorts: South Korea (n = 244,814) & Japan (n = 1,296,649)	6 ML models (ensemble)	ML outperformed classical statistical models in stability, generalizability, and reproducibility for 5-year incident HTN prediction	Yes (2 independent cohorts)	Not reported in abstract
Ye et al., 2018 [7]	Prospective EHR study	Statewide EHR cohort, Maine	XGBoost	AUC 0.870 in prospective cohort for 1-year incident HTN prediction (data leakage concern noted in published commentary)	Partial (prospective year)	No (data leakage concern noted)
Ambale-Venkatesh et al., 2017 [13]	Prospective cohort (MESA)	6814 adults, multi-ethnic, 12-year follow-up	Random survival forests	ML improved prediction of 6 CV outcomes vs. standard risk scores; imaging/ECG/biomarkers outperformed traditional risk factors	No independent external validation	Not reported
McManus et al., 2021 [23]	RCT (HOME BP)	622 patients, poorly controlled HTN	Digital/ML-assisted home BP monitoring	Significant SBP reduction vs. usual care in poorly controlled hypertension	Yes (RCT design)	N/A (RCT design)
Attia et al., 2019 [18]	Retrospective + prospective	44,959 patients	CNN applied to ECG	AI-ECG detected LV systolic dysfunction with high accuracy, outperforming standard ECG interpretation	Yes (prospective validation cohort)	Not reported in abstract
Kontopodis et al., 2023 [28]	Retrospective	Aortic aneurysm patients	ML (clinical + imaging variables)	ML improved AAA growth prediction incorporating clinical, biologic, morphologic, and biomechanical variables	No	Not reported
Weng et al., 2017 [29]	Retrospective cohort	378,256 UK Biobank patients	ML cardiovascular models	ML improved stroke and CHD prediction compared to established risk scores	Partial	Not reported

Abbreviations: AI = artificial intelligence; ML = machine learning; HTN = hypertension; CV = cardiovascular; EHR = electronic health record; ECG = electrocardiogram; CNN = convolutional neural network; SBP = systolic blood pressure; BP = blood pressure; AAA = abdominal aortic aneurysm; AUC = area under the curve; MESA = Multi-Ethnic Study of Atherosclerosis; RCT = randomized controlled trial.

**Table 2:** AI-Enabled Quality Improvement Interventions in Hypertension Care.

QI Target	AI Function	Mechanism of Action	Expected Clinical Impact	Deployment Stage	Implementation Considerations
Therapeutic inertia	Clinical decision support	Real-time treatment prompts	Improved BP control	Pilot/Early deployment	Avoid alert fatigue
Care gap detection	EHR-based ML screening	Identify uncontrolled patients	Earlier intervention	Research/Pilot	Workflow integration required
Risk-based triage	Predictive modeling	Stratify by organ damage risk	Targeted escalation	Research stage	External validation needed
Population health monitoring	Automated dashboards	Continuous performance tracking	System-level BP improvement	Early deployment	Data interoperability
Guideline adherence	Hybrid rule-based + ML	Compare practice to targets	Reduced variation	Pilot/Research	Clinician trust required
Outcome monitoring	Longitudinal analytics	Link processes to events	Organ protection focus	Research stage	Robust outcome data needed

Abbreviations: AI = artificial intelligence; QI = quality improvement; ML = machine learning; EHR = electronic health record; BP = blood pressure.

**Table 3:** Mechanistic Taxonomy: Data Modalities, Pathophysiological Pathways, and AI Evidence by Organ System.

End-Organ Target	Primary Pathophysiological Mechanism	Most Informative Data Modalities	AI Methodology	Current Evidence Stage
Hypertensive heart disease	Pressure overload → LV remodeling, myocardial fibrosis	ECG, echocardiography, BP variability metrics	CNN (ECG/imaging), temporal ML	Prospective validation (e.g., Attia et al. [18])
Aortic disease	Wall stress → medial degeneration, elastin fragmentation	CT/MRI morphology, biomechanical indices, BP burden	Imaging CNN, ML (clinical + imaging)	Retrospective; limited prospective data [28]
Hypertensive nephropathy	Microvascular injury → glomerulosclerosis, GFR decline	Serial eGFR, albuminuria, RAS medication history	ML regression, EHR-based models	Early clinical integration [47]
Cerebrovascular disease	Large-vessel atherosclerosis + small-vessel disease	BP variability, AF detection (ECG/wearable), imaging (WMH), carotid US	Multimodal ML, temporal modeling	Retrospective cohort studies [29]
Hypertensive retinopathy	Arteriolar narrowing, AV nicking, hemorrhage	Retinal fundus imaging	Deep learning (CNN)	Research stage; strong discriminative performance

Abbreviations: LV = left ventricular; BP = blood pressure; CNN = convolutional neural network; ML = machine learning; CT = computed tomography; MRI = magnetic resonance imaging; GFR = glomerular filtration rate; eGFR = estimated GFR; RAS = renin-angiotensin system; AF = atrial fibrillation; ECG = electrocardiogram; WMH = white matter hyperintensities; AV = arteriovenous; US = ultrasonography; EHR = electronic health record.

**Table 4:** Major barriers and enabling strategies for implementing AI-driven hypertension programs.

Domain	Barrier	Potential Risk	Mitigation Strategy
Model validity	Limited external validation	Reduced generalizability	Multicenter validation
Algorithmic bias	Data disparities	Health inequities	Subgroup reporting & recalibration
Workflow integration	Poor EHR integration	Alert fatigue	Clinician co-design
Interpretability	Black-box models	Reduced trust	Explainable AI methods
Data quality	Missing BP data	Model degradation	Standardized data capture
Regulatory oversight	Evolving governance	Safety concerns	Post-deployment monitoring
Privacy & security	Device integration risks	Loss of trust	Strong data governance
Interoperability	HL7-FHIR variability across EHR systems	Deployment failure across institutions	Standardized API conformance requirements; cross-system validation

Abbreviations: AI = artificial intelligence; EHR = electronic health record; BP = blood pressure; HL7 = Health Level 7; FHIR = Fast Healthcare Interoperability Resources; together, HL7-FHIR is a standard framework for exchanging electronic health information between systems.

## Data Availability

The data that support the findings of this study are available from the corresponding author, upon reasonable request.
